# Serum Thiols as a Biomarker of Disease Activity in Lupus Nephritis

**DOI:** 10.1371/journal.pone.0119947

**Published:** 2015-03-23

**Authors:** Pritesh Lalwani, Giselle Katiane Bonfim Bacelar de Souza, Domingos Savio Nunes de Lima, Luiz Fernando Souza Passos, Antonio Luiz Boechat, Emerson Silva Lima

**Affiliations:** 1 Programa de Pós-Graduação em Ciências Farmacêuticas (PPGCF), Universidade Federal do Amazonas, Manaus, Brazil; 2 Centro de Pesquisa Leônidas e Maria Deane (CPqL&MD)-FIOCRUZ, Amazônia, Manaus, Brazil; 3 Faculdade de Ciências Farmacêuticas, Universidade Federal do Amazonas, Manaus, Brazil; 4 Escola Superior de Ciências da Saúde, Universidade do Estado do Amazonas, Manaus, Brazil; 5 Faculdade de Medicina, Universidade Federal do Amazonas, Manaus, Brazil; 6 Instituto de Ciências Biológicas, Universidade Federal do Amazonas, Manaus, Brazil; Instituto Nacional de Ciencias Medicas y Nutricion Salvador Zubiran, MEXICO

## Abstract

Lupus Nephritis (LN) develops in more than half of the Systemic Lupus Erythematous (SLE) patients. However, lack of reliable, specific biomarkers for LN hampers clinical management of patients and impedes development of new therapeutics. The goal of this study was to investigate whether oxidative stress biomarkers in patients with SLE is predictive of renal pathology. Serum biochemical and oxidative stress markers were measured in patients with inactive lupus, active lupus with and without nephritis and compared to healthy control group. To assess the predictive performance of biomarkers, Receiver Operating Characteristic (ROC) curves were constructed and cut-offs were used to identify SLE patients with nephritis. We observed an increased oxidative stress response in all SLE patients compared to healthy controls. Among the several biomarkers tested, serum thiols had a significant inverse association with SLE Disease Activity Index (SLEDAI). Interestingly, thiols were able too aptly differentiate between SLE patients with and without renal pathology, and serum thiol levels were not affected by immunosuppressive drug therapy. The decreased thiols in SLE correlated significantly with serum creatinine and serum C3 levels. Further retrospective evaluation using serum creatinine or C3 levels in combination with thiol’s cutoff values from ROC analysis, we could positively predict chronicity of renal pathology in SLE patients. In summary, serum thiols emerge as an inexpensive and reliable indicator of LN, which may not only help in early identification of renal pathology but also aid in the therapeutic management of the disease, in developing countries with resource poor settings.

## Introduction

Systemic lupus erythematous (SLE) is a multisystem autoimmune inflammatory disease for which etiology and pathogenesis are incompletely understood [1]. However, multiple factors are thought to contribute to the development of immune response to self, including genetic, hormonal, and environmental factors [2]. Also, infectious pathogens have been suspected as cause and contributors of SLE, though direct evidence for their association is lacking [3–5]. The highest incidence of SLE is reported from tropical Brazil, and appears to be increasing as the disease is recognized more readily and patient survival rate increases [6].

One or more forms of glomerulonephritis develop in more than half the patients with SLE [7,8]. Evaluation for lupus nephritis (LN) includes urine sediment analysis, urinary protein and creatinine excretion, determination of serum creatinine and assessment of serological markers, such as anti-dsDNA antibody titers and, C3 and C4 complement levels [9,10]. LN is treated depending on the pathologic lesion with a combination of corticosteroids and immunosuppressive agents, particularly cyclophosphamide, azathioprine, or mycophenolate mofetil [8]. The principal goal of therapy in LN is to normalize renal function or, at least, to prevent the progressive loss of renal function. Although the use of aggressive immunosuppression has improved patient survival over the past several decades, managing relapses or flares requires constant follow-up and surveillance, which often entails changing treatments and remains challenging in LN [11].

Oxidative stress is increased in SLE, and it contributes to immune system dysfunction, abnormal activation and processing of cell-death signals, autoantibody production and fatal comorbidities [12,13]. Excessive reactive oxygen species (ROS) formation can induce oxidative stress; therefore, cells have antioxidant networks to scavenge excess ROS [12,14]. Mutation in NRF2 gene, a transcription factor of the antioxidant response pathway was associated with risk of nephritis in SLE patients [15]. Moreover, elevated serum nitrate and nitrite levels [16], homocysteine metabolism [17] and serum protein oxidation [18] has also been shown to be associated with disease activity and tissue damage in SLE. Also, increased malondialdehyde (MDA) a lipid peroxidation marker [19–21] and altered antioxidant enzymes, superoxide dismutase (SOD), catalase (CAT) and glutathione peroxidase (GPx) in the patients with SLE [19,20,22] has been described, confirming the altered oxidative stress responses in SLE [23]. Furthermore, higher SLEDAI is associated with lower serum albumin levels in LN [24,25]. However, currently there are no serum biomarkers of oxidative stress in routine clinical use.

Therefore, the purpose of this study was to determine whether serum oxidative stress biomarkers could be used as a marker of renal disease activity in SLE, which may not only have implications in better understanding of pathology but also in the therapeutic management of the disease.

## Materials and Methods

### Ethical Considerations

This study was approved by the Universidade Federal do Amazonas (UFAM) Research Ethics Committee, in accordance with Brazilian law, which complied with the Declaration of Helsinki. All the study participants provided signed an informed consent prior to enrolment.

### Patient samples

All study participants attended SLE clinic at the Ambulatório Araújo Lima, Hospital Universitário Getúlio Vargas (HUGV) [Manaus, Brazil] from September 2011 to June 2012. All the patients with SLE met the American College of Rheumatology (ACR) classification criteria for SLE [26]. For the purpose of this study patients were classified accordingly, (a) Lupus nephritis (LN) (SLEDAI >0): SLE patients were diagnosed for lupus nephritis based on blood test, urine analysis and kidney biopsy; 16 (52%) of LN patients were classified using microscopic analysis of urinary sediments, 24 hour proteinuria, serum creatinine and complement C3 levels, and 15 (48%) were classified using renal biopsies as per the International Society of Nephrology (ISN) and WHO criteria for SLE nephritis [27]; (b) Active lupus (AL) (SLEDAI >0): patients with active SLE without clinical or laboratorial signs of SLE nephritis. Enrolled patients had mucocutaneous manifestations such alopecia, malar rash or oral ulcers, arthritis or arthralgia, pleural effusion, hemolytic anemia, anti-phospholipid syndrome or other clinical signs or evidence of active disease, excluding nephritis; (c) Inactive lupus (IL) (SLEDAI = 0): inactive SLE patients without nephritis. The assessment of disease activity was performed using SLEDAI calculation for each patient [28]. In addition, a group of healthy individuals was recruited as a control group. Patients with diabetes or other metabolic diseases, end-stage renal disease, smoking, pregnant women and individuals with HIV or HCV infection were excluded from this study.

Ninety-three consecutive patients were enrolled to participate in the study after signing the consent form. Venous blood was collected from SLE patients and healthy controls and split into two parts: one part was used immediately for hematological analysis to avoid contamination and hemolysis, whereas, the second part was immediately centrifuged at 2000*g*, 10 minutes, the plasma or serum was decanted and stored at −70°C until analysis.

In addition, from the patients’ medical records following information was gathered and collated on a form: disease duration, time of lesion, age at onset; comorbidities such as hypertension and SLE manifestation’s such as Raynaud, vasculitis, pulmonary hypertension and antiphospholipid syndrome; Anti nuclear antibodies (ANA), anti-dsDNA, anti-Sm, anti-cardiolipin (CL), anti-Ro and anti-La antibodies; SLE nephritis criteria at time of assessment, biopsy activity and index scores and urine analysis; immunosuppressive drugs being used.

### Serum biomarkers

Quantification of the complement fractions C3 and C4 was performed by turbidimetry method using the COBAS Mira Plus Analyzer with appropriate kits provided (Human GmbH, Germany) following the manufacturer’s instructions. Serum creatinine, urea, uric acid, total proteins were also quantified using COBAS Mira Plus Analyzer.

Quantitative measurement of serum albumin, thiols, MDA, GPx and GR was performed as described previously [21,29,30]. For Allantoin, CAT, total antioxidant capacity, total ROS stress index the measurements was performed using established methods (Young & Conway 1942, Aebi 1984, Erel 2004, Aycicek et, al. 2005). Superoxide Dismutase (SOD) assay was performed using Ransod SD125 kit (Randox) following the manufacturer’s instructions.

### Sample size and power analysis

For sample size calculation and power analysis we chose the total serum thiols as the main study variable (dependent variable). From our previous results [21], we observed a detectable difference of 80μmol/L and a standard deviation of 50μmol/L in serum thiol levels. Setting a significance level of 0.05 and power at 80%, we calculated an effect size of 1.32 and estimated a sample size of 12 patients for each study group. Sample size calculation and power analysis was performed using the GPower 3.1 software [31].

### Data analysis

Descriptive statistics was used to describe the main clinical and demographic SLE features. The two-tailed Student t-test or One-Way ANOVA was used to compare means when normal distribution was observed in a Kolmogorov-Smirnov test. When normality was not observed the non-parametric Mann-Whitney test was used to compare medians. For ANOVA post-hoc analysis the Tukey test was used for intergroup assessment. Linear Regression was performed to assess the relation between variables. Normality for regression analysis was assessed using D’Agostino and Pearson normality test. ROC curve analysis was performed to evaluate each oxidative stress or inflammatory marker. Significance level adopted was 0.05 and all tests were two-tailed. All above-mentioned statistical tests were performed using the Prism 6.02 software (GraphPad).

## Results

### Oxidative stress is elevated in SLE patients

The demographic characteristics and drugs used by different groups included in this cross sectional study are presented in Table 1. Disease activity was determined by using SLEDAI score. LN patients had a significantly higher SLEDAI score compared to AL patient group. LN patients (n = 31) were identified by clinical and laboratorial diagnosis, 15 (48%) of the biopsied patients had a mean chronicity index ranged from 2.54 to 4.81 and the mean activity index ranged from 8.65 to 11.89; the patients included in the study had class type II (n = 1), type III (n = 9), type IV (n = 20), type V (n = 1) grade of disease according to the International Society of Nephrology (ISN) criteria for SLE nephritis classification.

**Table 1:** Demographic characteristics and medication of SLE patients.

	**Healthy controls (C)**	**Inactive Lupus (IL)**	**Active Lupus (AL)**	**Lupus Nephritis (LN)**	**P value**
Total number of patients	11	30	21	31	
Age (years)	32.73 ±8.86	31.67 ±7.91	30.05 ±5.97	28.29 ±6.06	0.331
Duration of disease (months)		97.7 ±61.36	52 ±50.89	53.27 ±55.37	**0.003**
Duration of lesion (months)			2.8 ±1.55	3.06 ±1.53	0.675
SLEDAI (score)			10 ±2.59	14.77 ±3.03	**<0.001**
Anti-dsDNA antibodies n (%)			16 (76)	26 (83)	
**Drugs** n (%)					
Prednisone		21 (70)	21 (100)	29 (94)	**0.007**
Chloroquine / hydroxychloroquine		23 (77)	17 (81)	19 (61)	0.149
Azathioprine		5 (17)	4 (19)	3 (10)	0.594
Methotrexate		1 (3)	2 (10)	1 (3)	0.551
Mycophenolate mofetil / sodium		1 (3)	3 (14)	8 (26)	**0.044**
Cyclophosphamide		0 (0)	1 (5)	13 (42)	**<0.001**
Methylprednisolone		0 (0)	2 (10)	5 (16)	0.080

Due to the multiplicity of symptoms and organ system involvement with SLE, its severity in an individual must be assessed in order to successfully treat. The treatment of SLE involves immunosuppressants to prevent flares and reducing their severity and duration when they occur. A higher usage of corticosteroid, prednisone was observed in AL and LN patients compared to IL patients. In addition, a greater use of mycophenolate mofetil/sodium and cyclophosphamide was observed in treating AL and NL group compared to IL patients. Our lupus nephritis patients were classified into two groups depending on the drugs prescribed, treatment regime A (azathioprine, chloroquine/hydroxychloroquine, methotrexate and prednisone) and B (cyclophosphamide, methylprednisolone and mycophenolate mofetil/sodium). Individuals with treatment regime A (n = 10) and B (n = 21) had 50% and 70% LN class type IV patients, respectively.

To comprehend the role of oxidative stress in the progression of SLE, the level of multiple renal function and oxidative stress markers were measured in SLE patients and healthy controls. Table 2 depicts the level of serum biomarkers in different study groups. Serum creatinine is the most widely used screening test to identify abnormalities in renal function; we observed increased levels in LN (1.27±0.41) compared to control (C) (0.60±0.13), IL (0.61±0.16) and AL (0.59±0.18) groups. Furthermore, we also observed significantly altered levels of complement C3 (p<0.001) and C4 (p = 0.017) proteins as demonstrated previously to be altered in kidney disease. In comparison to creatinine, C3 levels in serum decreased in LN (74.13±58.08) patients in comparison to C (168.10±39.66), IL (143.90±50.11) and AL (101.20±77.64) patient groups. Also, urea (p<0.001) and uric acid (p<0.001) levels were significantly elevated in LN patients compared to other study groups.

**Table 2:** Comparison of serum biomarkers in SLE and healthy subjects.

	**Healthy controls (C)**	**Inactive Lupus (IL)**	**Active Lupus (AL)**	**Lupus Nephritis (LN)**	**P value**
Albumin (g/dL)	4.99 ±0.25	4.51 ±0.36	4.23 ±0.52	3.47 ±0.60	**<0.001**
C3 (g/dL)	168.10 ±39.66	143.90 ±50.11	101.20 ±77.64	74.13 ±58.08	**<0.001**
C4 (g/dL)	30.27 ±14.91	23.27 ±10.70	16.52 ±12.25	24.26 ±11.43	**0.017**
Creatinine (mg/dL)	0.60 ±0.13	0.61 ±0.16	0.59 ±0.18	1.27 ±0.41	**<0.001**
Total Protein (g/dL)	7.01 ±0.35	6.48 ±0.66	7.01 ±0.82	5.59 ±0.85	**<0.001**
Urea (mg/dL)	28.45 ±6.41	31.87 ±5.38	37.80 ±9.66	67.40 ±43.26	**<0.001**
Uric Acid (mg/dL)	4.29 ±1.10	4.58 ±0.99	5.78 ±1.84	7.40 ±2.29	**<0.001**
Allantoin (mg/L)	3.43 ±0.83	5.95 ±1.93	5.89 ±1.90	7.61 ±3.21	**<0.001**
CAT (k/gHb/min)	2.74 ±1.50	2.25 ±0.79	3.09 ±2.20	2.97 ±1.76	0.642
GPx (U/L)	4436.49 ±1142.00	5893.00 ±1710.68	4183.41 ±930.56	4859.97 ±1363.30	**<0.001**
GR (U/L)	27.59 ±17.71	22.57 ±11.51	38.51 ± 23.41	31.97 ±18.32	**0.028**
MDA (μol/L)	4.03 ±0.95	3.37 ±1.06	5.41 ±1.31	3.81 ±1.42	**<0.001**
SOD (U/mL)	684.64 ±191.47	687.40 ±260.74	654.43 ±179.07	621.43 ± 244.55	0.784
Oxidative stress index	0.84 ±0.30	1.45 ±0.93	2.17 ±1.96	1.01 ±0.81	**0.001**
TAC (mmol/L)	1.00 ±0.10	0.84 ±0.16	0.68 ±0.27	0.93 ±0.25	**<0.001**
Total ROS (μmol/L)	8.29 ±2.94	11.35 ±5.93	10.75 ±5.25	7.58 ±3.90	**<0.001**
Thiols (μmol/L)	262.80 ±63.17	256.40 ±65.06	133.50 ±62.17	84.50 ±36.55	**<0.001**

Catalase (CAT), Glutathione peroxidase (GPx), Glutathione reductase (GR), Malondialdehyde (MDA), Superoxide dismutase (SOD), Total antioxidant capacity (TAC), Total reactive oxygen species (ROS).

Oxidative stress markers in lupus patients were significantly altered compared to controls as depicted in Table 2, analyzed by Kruskal Wallis test. Allantoin a product of oxidation of uric acid by purine catabolism was significantly (p<0.001) altered in lupus patients, this is in accordance with the uric acid levels (Table 2). Also, GPx (p<0.001), glutathione reductase (GR) (p = 0.028) levels were also altered in the study groups. Lipid peroxidation marker, MDA (p<0.001) was also significantly altered in the lupus patients. On the other hand CAT and SOD levels were unaltered in the different lupus patients. However, total serum thiol and total ROS levels were significantly altered in the study groups. In addition, a statistically significant altered oxidative stress index and total antioxidant capacity was observed in the lupus patients compared to control. Next, we analyzed AL group with LN group with Mann Whitney U test to identify biomarkers that can differentiate these two patient groups. Thiols (p<0.0047), MDA (p<0.0001) and total ROS (p = 0.0008) levels were significantly decreased in LN compared to AL group (data not shown).

In summary, these results demonstrate an altered renal function and increased oxidative stress in patients with lupus nephritis.

### Thiols differentiate between lupus nephritis and other systemic lupus erythematous patients

**Fig 1:**
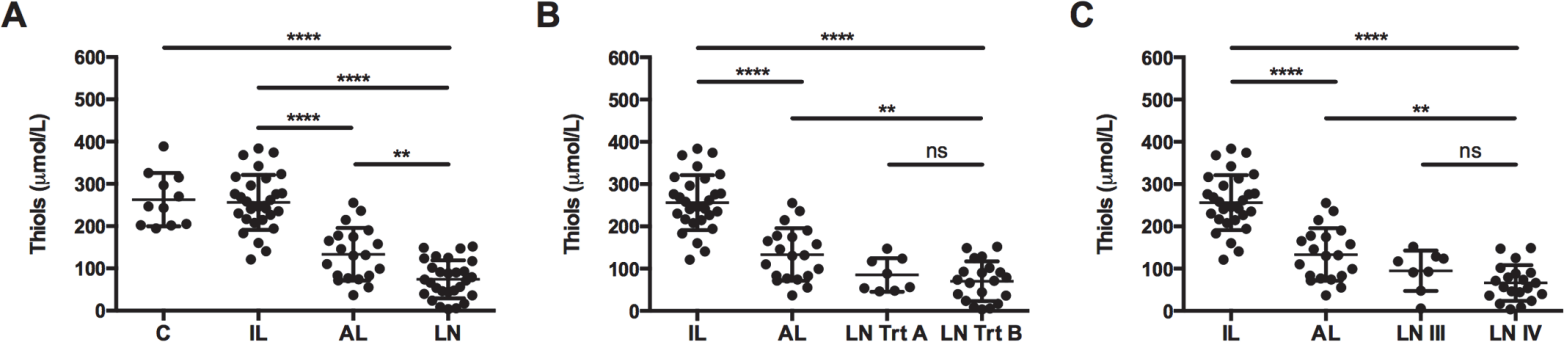
Serum thiols differentiate SLE patients with and without Lupus nephritis. (A) Serum thiols were measured in healthy controls [C], inactive lupus [IL], active lupus [AL] and lupus nephritis [LN] patients. (B) Thiol levels in LN patients with treatment A [Trt A] or treatment B [Trt B] was compared to IL and AL patient groups. (C) Lupus patients were classified into LN type III [LN III] or type IV [LN IV] depending on the severity of the disease and thiol levels were compared with IL and AL patients. Each dot represents data from an individual subject (**P<0.005; ***P <0.0001).

We evaluated if the oxidative stress markers can differentiate between inactive lupus, active lupus and patients with lupus nephritis patient groups. As mentioned previously, thiols, MDA and total ROS were able to differentiate between AL and LN patients. However, when we compared MDA and total ROS levels between healthy controls, IL, AL and LN groups, no significant differences were observed, which make it difficult to be used as a biomarker of disease activity (data not shown). On the other hand, serum thiols were able to significantly differentiate LN patients from controls, IL and AL patients (Fig. 1A). Next, we checked if thiols were influenced by use of corticosteroids and immunosuppressive drugs in nephritis patients. We observed no differences in thiol levels in patients with treatment regime A (Trt A) compared to treatment regime B (Trt B) (Fig. 1B). Mean thiol levels were the same in LN patients treated with Trt A or Trt B, suggesting corticosteroids and immunosuppressive drugs did not influence serum thiols. However, a significant difference was observed in IL and AL groups compared to LN patients with Trt B (Fig. 1B). Subsequently, we evaluated if this oxidative stress markers can differentiate LN disease severity. Class IV LN group had significantly lower thiol levels compared to IL and AL groups (Fig. 1C).

**Fig 2:**
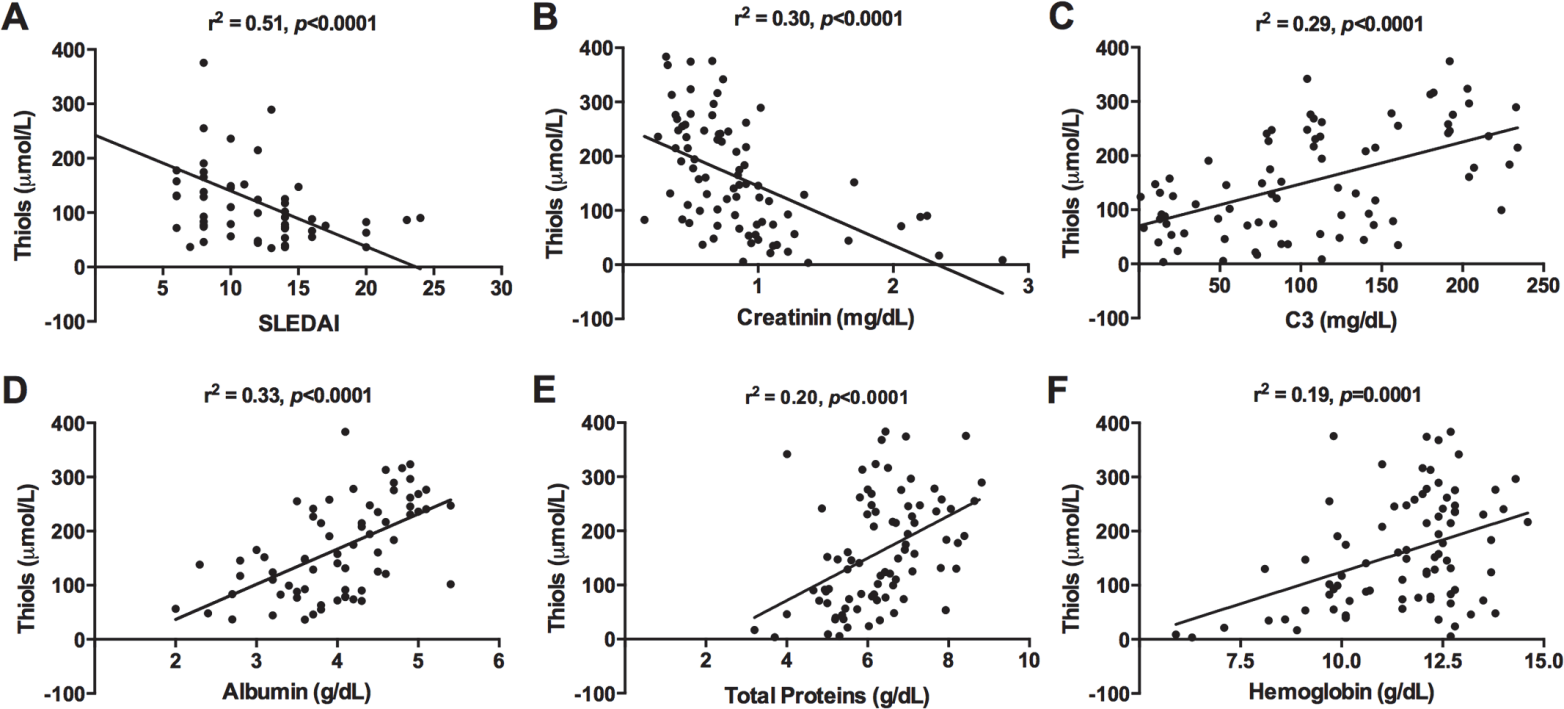
Serum thiol levels correlate with SLE disease activity. Correlation between serum thiols and (A) SLEDAI, (B) serum creatinine, (C) serum albumin, (D) complement protein C3, (E) total serum proteins and (F) hemoglobin levels was performed in SLE patients. The x-axis shows SLE disease markers and the y-axis indicates thiol levels. The correlation coefficient (r^2^) and P value is mentioned for each graph.

Next, we formed a correlation analysis of thiols with other SLE biomarkers (Fig. 2). A significant and strong negative correlation of thiols was observed between SLEDAI (r^2^ = 0.51, p<0.0001), which is in line with previous observations that there is a decrease in serum thiol levels with disease progression. Also, serum creatinine had a significant but weak negative correlation with thiols, increasing level of creatinine is directly proportional to decreasing level of thiols. On the other hand, serum albumin and C3 demonstrated a positive correlation with thiols. We also observed a negative correlation when we compared SLEDAI to albumin and C3 (data not shown). Decrease in serum thiols is associated with decreased total serum proteins in SLE patients.

Moreover, when we performed a post-hoc power analysis using results showed in Table 2, we observed that this study achieved at least 99% of statistical power (data not shown). As a consequence, our sample was large enough to confirm our hypothesis that serum thiols are lower in class IV nephritis patients compared to other SLE groups. In short, serum thiols not only correlate with disease activity in lupus patients but also differentiate between lupus nephritis and other SLE. They also correlate well with current clinically used markers for LN and was not influenced by drugs.

### Serum thiols as a marker of lupus nephritis activity

**Fig 3:**
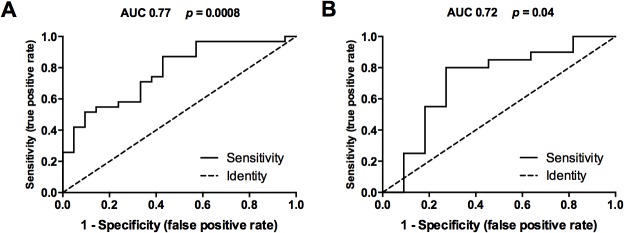
Sensitivity and specificity profile of thiols as a biomarker in SLE. Receiver operator curve (ROC) analysis was performed to study predictive performance of serum thiols (A) patients with active lupus (n = 21) from lupus nephritis (n = 31) and (B) patients with lupus nephritis non-type IV (n = 11) from type IV (n = 20). The ROC curves plot (1-Specificity) % on the x-axis versus the Sensitivity (%) on the Y-axis. AUC = Area under ROC curve and P value is mentioned for each graph.

Serum thiol levels were used for ROC curve (Fig. 3A) analysis, AL group v/s LN group, which had an area under curve (AUC) of 0.77, and a sensitivity of 80% and specificity of 60% with a cutoff ≤124 μmol/L (Fig. 3A). However, ROC analysis with LN non-class IV v/s class IV, an AUC of 0.72 and cutoff ≤90.90 μmol/L, was observed (Fig. 3B). Furthermore, a sensitivity of 80% and an improved specificity of 72%, demonstrated that thiols is a reliable marker to discriminate patients with nephritis disease activity.

Next, we retrospectively analyzed our patient group if we could identify ones with LN type class IV using thiols as a biomarker (Table 3). Currently, creatinine and C3 are routinely used biomarkers for kidney diseases. We used contingency table and performed a fishers exact test to test the hypothesis, if a patient with SLE tests for thiols (cutoff ≤90.90 μmol/L), creatinine (cutoff >1.10 mg/dL) and C3 (≤100 g/dL), can we predict whether the patient has LN with class IV. Use of serum creatinine and thiol measurement in an SLE patient was significantly associated with the identification of LN type IV patients (p<0.0002) (Table 3). However, if we measured thiols and C3, the probability of identifying a patient with LN type IV was significantly increased (p<0.0001) (Table 3).

**Table 3:** Retrospective identification of Lupus nephritis class IV patients.

	**LN Class IV**		
	**Yesn (%)**	**Non (%)**	**P value**
Creatinine	11 (55)	9 (45)	
Thiols	15 (75)	5 (25)	**0.0002**
Thiols and Creatinine	16 (80)	4 (20)	
C3	15 (75)	5 (25)	
Thiols	15 (75)	5 (25)	**0.0001**
Thiols and C3	19 (95)	1 (5)	

Cut-off: Creatinine >1.10 mg/dL; C3 <100 g/dL; Thiols <90.90 μmol/L

Collectively, we have demonstrated that serum thiols are suitable biomarkers to predict nephritis in SLE patients and also differentiated between the disease activities.

## Discussion

Glomerulonephritis develop in more than half of the SLE patients, which make monitoring of renal disease activity in patients of utmost importance. The present study found increased oxidative stress in SLE patients and an inverse association between serum thiols and disease activity assessed by SLEDAI index, where lower thiols were associated with higher level of SLE disease activity. Several recent studies have also demonstrated increased oxidative stress responses in SLE; however, no biomarkers of oxidative stress are yet in routine clinical use [12].

Patients with inactive lupus (IL) had a disease for longer duration than patients with active lupus (AL) and lupus nephritis (LN), suggesting that some genetic factors might drive the disease outcome. However, ROS generated by macrophage and neutrophils play a major role in inflammation and cell death that characterize SLE pathology [12,32]. Thiols constitute a major portion of the total body antioxidants and they play a significant role in defense against ROS [33]. Among the thiols that are bound to proteins, human serum albumin makes the major portion of the protein bound thiols [34,35]. In our study, decreased serum albumin [25] and total thiols in SLE was observed, corroborating with previous results. The most striking result to emerge from this analysis was that thiol levels could differentiate between SLE patients with and without lupus nephritis. Furthermore, thiol levels were not perturbed by chemotherapeutic and immunosuppressive drug use, which makes it an attractive candidate to be used as a biomarker.

Additionally, if free radical species are not scavenged, they can induce lipid peroxidation and tissue damage. The analysis showed that total ROS was significantly increased in AL compared to healthy controls; in contrast, it was decreased in LN compared to AL. It has been demonstrated that cyclophosphamide can induce [36], whereas, mycophenolate can inhibit ROS production [37,38] in several cell types. Similarly, MDA a marker of lipid peroxidation was increased in AL compared to healthy controls; on the other hand, it was reduced in LN compared to AL. Cyclophosphamide has been shown to induce [39] and mycophenolate to inhibit MDA expression [40]. Even though our results for LN might differ from some previous studies [20,41], the trend is similar for MDA and ROS, nevertheless, we demonstrate increased allantoin production as a marker of free radical generation in AL and LN patients compared to control group, suggesting that overall drug usage may have contributed to these discrepancies. Further investigation is required to understand the impact of drugs and oxidative stress on disease outcome in SLE.

Uric acid is the final product of purine metabolism; it reacts with free radicals to produce allantoin. Uric acid and urea levels were significantly altered in SLE and more so in LN [10]. Overall allantoin levels were also elevated in SLE patients compared to control group, in accordance with previous reports [42]. Enzymes GPx, GR, SOD and CAT possess the ability to scavenge reactive oxygen intermediates and protect against oxidative damage [12,43]. Though, GPx activity was decreased in AL group compared to controls; on the other hand LN had increased levels of GPx compared to control. Previously, Shah et.al observed decreased GPx levels in SLE patients [19,20,41]. But, cyclophosphamide use upregulates GPx expression [44], which may be the reason for the observed differences. Furthermore, a modest increase of GR was observed in SLE patients compared to control group.

On the contrary, serum SOD was not altered in our study groups, however, several studies have demonstrated unchanged, increased or decreased levels in SLE [14,19,20,45]. Similarly, CAT was also not significantly altered in SLE group, whereas, increased and decreased activity of CAT in SLE has been reported [19,20,46]. These differences in SOD and CAT activities could be due to the presence of antibodies against these enzymes, which may form immune complexes and reduce enzyme activity [46]. Overall, we observed a decreased antioxidant capacity and an increased oxidative stress index in SLE patients.

Currently, anti-DNA antibody titers, serum creatinine and complement levels are widely used as a marker for renal disease activity but, these serologic parameters are not specific and sensitive for this manifestation and their performance as nephritis biomarkers is not optimal [11].

In our study, we found strong and statistically significant associations between serum thiols, and SLEDAI and serum creatinine in SLE patients. Also, association between thiols and albumin, C3, total proteins and hemoglobin was weak but statistically significant. Anemia is common in about chronic renal failure, however, role of thiols if any is unclear [47]. Previously, serum albumin was shown to correlate with disease activity and was suggested as a screening marker [25] however, we observed that the dynamic range of change observed in serum albumin levels in SLE is very small, thus measuring of total serum thiols would be more advantageous.

A retrospective analysis using cutoff values from ROC curves could positively identify SLE patients with nephritis. Surprisingly, measuring serum creatinine or C3 in combination with serum thiols significantly increased the association with nephritis and was able to accurately identify LN type IV patients. It was our objective to determine whether serum thiol level correlates with SLE disease activity. Our study in fact showed a strong inverse relationship between thiols and SLEDAI in patients with nephritis. Moreover, we could demonstrate that measuring serum thiols in combination with serum creatinine or C3 may be an excellent screening test to detect SLE patients with renal involvement, which would prompt the ordering of a 24-hour urine collection for proteinuria or a biopsy. Our study has some limitations. We cannot rule out the possibility that other SLE patients without nephritis had a mild form of renal diseases, however, we did not observe any clinical or altered laboratory renal markers in these patients. Furthermore, we don’t know, which thiols are decreased in SLE patients other than serum albumin, and the mechanisms involved. However, we observed that quantification of total thiols provides a dynamic range for differences between SLE patients and it is inexpensive compared to measuring only one thiol type. The sample size was small to draw firm conclusions, besides, use of thiols, as a biomarker needs to be evaluated in a larger sample. Additionally, a follow-up cohort study can help us better comprehend if thiols can be used as marker for nephritic flares.

In summary, we demonstrated that serum thiols are inversely associated with SLE disease activity and this association was stronger in those with lupus nephritis. The clinical implications of these results are that serum thiols will alone not be the ideal biomarker to identify disease activity in SLE. It may, however, be a useful screening test and guide in combination with conventional tests in developing countries with resource poor settings. Future studies are needed to assess the clinical utility of serum thiols as a marker not only for a better understanding of renal pathology but also in the therapeutic management of the disease.
